# Management of Bivalirudin Anticoagulation Therapy for Extracorporeal Membrane Oxygenation in Heparin-Induced Thrombocytopenia: A Case Report and a Systematic Review

**DOI:** 10.3389/fphar.2020.565013

**Published:** 2020-09-11

**Authors:** Han Zhong, Ming-Li Zhu, Yue-Tian Yu, Wen Li, Shun-Peng Xing, Xian-Yuan Zhao, Wei-Jun Wang, Zhi-Chun Gu, Yuan Gao

**Affiliations:** ^1^ Department of Pharmacy, Renji Hospital, School of Medicine, Shanghai Jiaotong University, Shanghai, China; ^2^ Department of Critical Care, Renji Hospital, School of Medicine, Shanghai Jiaotong University, Shanghai, China; ^3^ Department of Cardiovascular Surgery, Renji Hospital, School of Medicine, Shanghai Jiaotong University, Shanghai, China

**Keywords:** bivalirudin, anticoagulants, extracorporeal membrane oxygenation, heparin-induced thrombocytopenia, management strategy

## Abstract

Extracorporeal membrane oxygenation (ECMO) can provide respiratory and cardiac support to patients in reversible devastated conditions. Heparin is the mainstay for anticoagulation during ECMO. Bivalirudin, a direct thrombin blocker, may represent an effective alternative for patients suffering from heparin-induced thrombocytopenia (HIT). We present the first case of a Chinese patient who experienced HIT and received bivalirudin anticoagulation during ECMO. In addition, we present a systematic review for this topic. We searched PubMed, EMBASE, and Cochrane Library (up to April 20, 2020) for studies that included patients undergoing ECMO, presenting with HIT, requiring bivalirudin treatment, and reporting relevant outcomes. The literature review yielded 15 studies involving 123 patients, amongst whom 58 patients were confirmed or suspected HIT patients, and 76 patients received bivalirudin as an anticoagulant for ECMO. Twelve studies were included for quantitative synthesis, and 46 patients were retrieved. The mean age of these patients was 46 years, and 30 patients were males. The average maintenance rate of bivalirudin was 0.27 ± 0.37 mg/kg/h, in order to maintain a target of activated clotting time (ACT) of 160–220 s. Additionally, bivalirudin doses in patients with continuous renal replacement therapies (CRRT) and patients without CRRT were 0.15 ± 0.06 mg/kg/h vs 0.28 ± 0.36 mg/kg/h, respectively (*p*=0.15). Most of the patients with confirmed HIT improved platelet counts in 3.3 ± 2.8 days after switching to bivalirudin anticoagulation. The patient-level data showed that 29 cases survived, 1 reported major bleeding, and 4 reported thrombotic events. Bivalirudin might be a promising optimal choice for ECMO anticoagulation in patients with HIT. A tailored protocol for management of bivalirudin treatment during ECMO should be developed with caution. Further prospective studies are necessary to standardise the use of bivalirudin.

**Systematic Review Registration:**

PROSPERO, identifier CRD42020160907.

## Background

Extracorporeal membrane oxygenation (ECMO), including venovenous (VV)-ECMO and venoarterial (VA)-ECMO, is an important circulatory support utilised in critically ill populations with reversible respiratory and/or cardiac failure (Macielak et al., 2019; Pollak, 2019). Although ECMO is life-saving, this intervention mandates the risk of thrombotic complications because of the continuous interaction between blood and artificial surfaces within the extracorporeal circuit (Laurance Lequier et al., 2014). Therefore, exogenous anticoagulant supplementation is necessary.

Unfractionated heparin (UFH) is the first choice for ECMO systematic anticoagulant because it is cheap, feasibly titratable, and easily reversible by protamine (Garcia et al., 2012; Koster et al., 2019). However, the application of UFH induces heparin-induced thrombocytopenia (HIT), which is a potentially fatal immune disorder and manifests reduced platelet counts (approximately 5–10 days after UFH exposure) with or without thrombosis (Martel et al., 2005). Generally, the incidence of HIT is approximately 0.1%–5% (Bruno et al., 2003; Pollak et al., 2011). HIT prevalence is even higher in patients with prolonged anticoagulation during ECMO (Laverdure et al., 2016). Therefore, the treatment of HIT requires instant alteration of anticoagulation from UFH to other agents (Koster et al., 2007).

Bivalirudin, an inhibitor with intermediate affinity to thrombin, can provide an appealing choice because of its unique pharmacological profiles (Richard et al., 2010). Firstly, bivalirudin can directly inhibit plasma thrombin, clot-bound thrombin, and collagen-induced platelet activation without the cofactor antithrombin III (Erdoes et al., 2019; Walker et al., 2019). Secondly, bivalirudin has a short half-life of approximately 25 min because it is mainly metabolised by proteolysis (Berei et al., 2018). Thirdly, the anticoagulant efficacy of bivalirudin can be monitored by activated clotting time (ACT) and activated partial thromboplastin time (APTT) which show good correlation (Casserly et al., 2004; Pollak, 2018). However, the standardised management of bivalirudin administration in ECMO patients with HIT has not been clearly elucidated due to limited data.

We present the first report of a Chinese female on ECMO who switched to bivalirudin anticoagulation after occurrence of HIT. Besides, we present a systematic review of the studies reporting the dosage, monitoring, and clinical outcomes of bivalirudin administration in adult ECMO patients with HIT. Finally, we set up a standard management flowgram for such patients in order to share our experience of maintaining the clinical efficacy and safety of bivalirudin anticoagulation therapy during ECMO.

## Case Presentation

**Figure 1:**
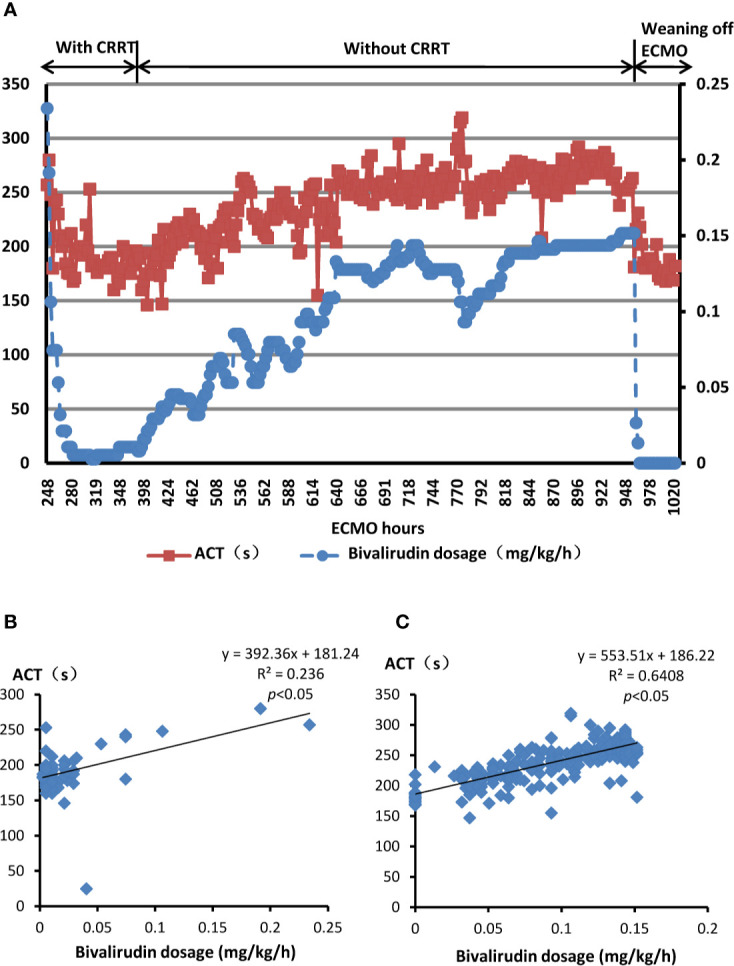
Bivalirudin dosing and relevant ACT in the present patient with HIT. **(A)** The dosage of bivalirudin and the ACT monitoring during bivalirudin treatment. Red squares indicate ACT values while the blue dos indicate bivalirudin doses. **(B)** The linear relationship between bivalirudin dosage and ACT value undergoing ECMO with CRRT. **(C)** The linear relationship between bivalirudin dosage and ACT value undergoing ECMO without CRRT. HIT, heparin-induced thrombocytopenia; ECMO, extracorporeal membrane oxygenation; CRRT, continuous renal replacement therapies; ACT, activated clotting times. y (dependent variable): ACT(s), x (independent variable): dosage of bivalirudin (mg/kg/hour).

**Figure 2:**
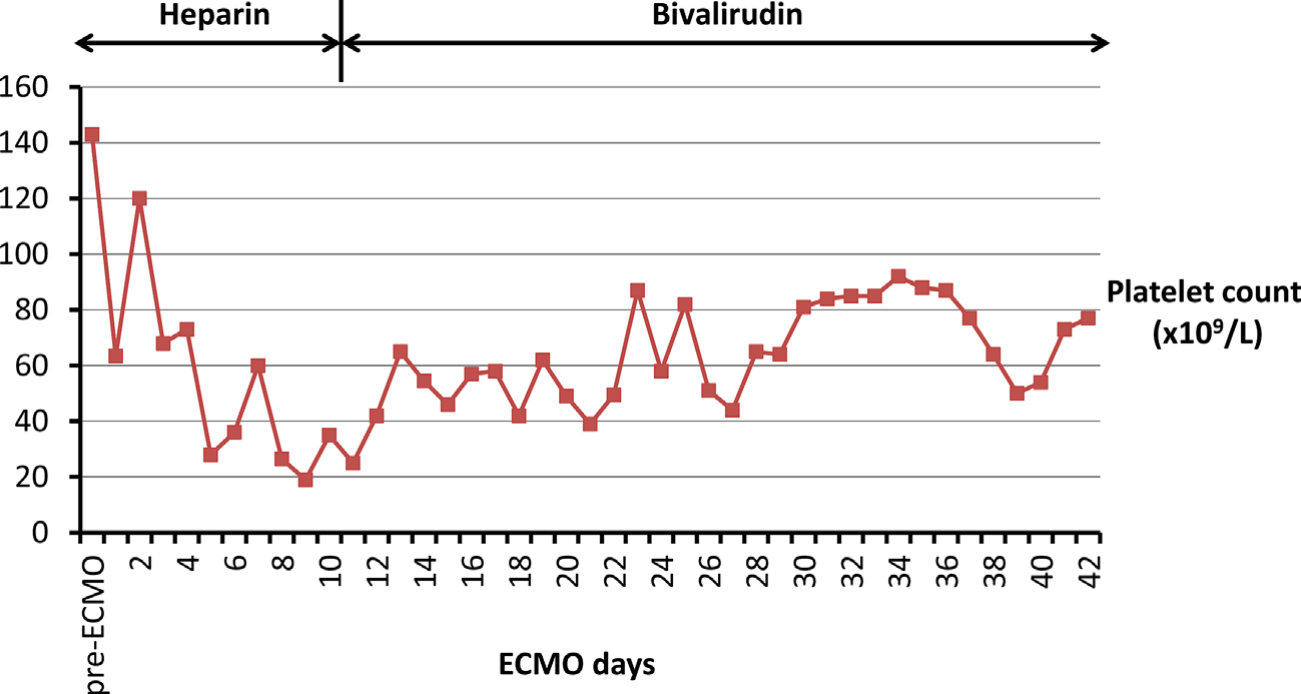
Platelet counts in the present patient with HIT are displayed during ECMO support with heparin therapy and bivalirudin anticoagulation, respectively. The red squares indicate platelet counts (*10^9^/L). HIT, heparin-induced thrombocytopenia; ECMO, extracorporeal membrane oxygenation.

In June 2019, a 27-year-old woman (weight 47 kg) with a 2-month history of exacerbating exercise intolerance and dyspnea was admitted to our hospital at 20 weeks pregnancy. Examination was unremarkable except tachypnea (30 breaths per min). Arterial blood gas analysis showed low partial pressure of oxygen (57.2 mmHg on room air). Her brain-type natriuretic peptide (BNP) level was 680 pg/ml, and troponin I level was 0.17 ng/ml. An electrocardiogram showed ST-T wave abnormalities. Transthoracic echocardiography displayed a dilated right ventricle (right ventricular internal diameter: 44 mm) with a “D-shaped” left ventricle, elevated systolic pulmonary artery pressure (154 mmHg), and patent ductus arteriosus. The diagnosis was pregnancy, severe pulmonary artery hypertension (PAH), cardiac failure (New York Heart Association III), and acute respiratory failure (type I). Her medication was started with 25 mg sildenafil, three times daily, and 1.25 ng/kg/min treprostinil. Treprostinil dose was progressively increased to 17 ng/kg/min. One week later, her pregnancy was terminated, and she was transferred to the intensive care unit. As a result of continuous deterioration, she experienced sudden cardiac arrest. Cardiopulmonary resuscitation was applied immediately along with invasive ventilation. Inotropic support with dopamine, epinephrine, and norepinephrine was initiated. A VA-ECMO support with a flow of 3–3.5 L/min was indicated simultaneously. The VA-ECMO consisted of a standard pump/oxygenation combination (BE-PLS 2050, MAQUET Cardiopulmonary GmbH, Baden-Württemberg, Germany) and inflow/outflow cannulas (CB96570/96670-015, Medtronic Inc., Michigan, USA). UFH with an infusion rate of 3–13 units/kg/h was used for anticoagulation to maintain a target ACT in the range of 180–220 s and APTT in a range of 35–75 s. In addition, ambrisentan at a daily dose of 5 mg was supplied to control PAH. Thereafter, the systolic pulmonary artery pressure was controlled between 86 to 96 mmHg, and normal ventricles were observed by echocardiography. On day 5 of ECMO, platelet count decreased abruptly from 147.5x10^9^/L to 28x10^9^/L. Platelet transfusion was performed, but the platelet counts further dropped to a nadir of 19x10^9^/L at day 9 of ECMO. HIT was therefore suspected, although the patient did not develop any thrombotic event. The pretest clinical scoring system, the 4T’s (Lo et al., 2006), was used to identify the possibility of HIT in the patient. The score was 5 points, indicating intermediate probability of HIT. Two days later (day 11 of ECMO), platelet factor 4 (PF-4)/heparin antibody titred by latex enhanced turbidimetric immunoassay (ACL-TOP700 automatic coagulation analyzer, Beckman Coulter, California, United States) returned to a positive result of 8.7 units/ml (normal range 0–1 units/ml). Thus, HIT was diagnosed in the patient. The patient received therapeutic plasma exchange with a flow of 1,200–1,500 ml/h for 2.5 h for PF-4/heparin antibody removal from her blood, despite plasma exchange was not a recommended treatment for HIT and it was occasionally used in patients with acute HIT (Cuker et al., 2018). Meanwhile, anticoagulation was then performed using bivalirudin (TAIJIANING; SALUBRIS Pharmaceuticals Co. Ltd., Shenzhen, China). Initially, bivalirudin was used with a dose of 0.23 mg/kg/h and was titrated according to ACT and APTT. The average dose of bivalirudin used was 0.005–0.03 mg/kg/h with continuous renal replacement therapies (CRRT) and 0.03–0.15 mg/kg/h without CRRT (**Figure 1A**). Target ACT (200–250 s) and APTT (40–60 s) were managed easily with bivalirudin and no supplemental boluses were needed (**Figure 1A**). The titre of anti-PF4/heparin antibody gradually reduced and returned to negative (0.3 units/ml) on the day 33 of UFH withdrawal. The platelet counts gradually increased and remained stable (70–90 x10^9^/L) throughout the infusion of bivalirudin (**Figure 2**). The patient was successfully weaned off ECMO after a total of 42 days. During the ECMO support procedure, 39 units of platelet concentrates, 59 units of red blood cells, and 7.6 L of fresh frozen plasma were transfused. The oxygenator was changed twice, while the circuits were changed once. Bivalirudin infusion was stopped 12 h later, and the ACT decreased to a value of 180 s within 4 h. The total duration of bivalirudin was 31 days. There was no significant bleeding and circuit obstruction during its use.

## Methods

### Electronic Searches

We searched PubMed, EMBASE, and the Cochrane Library from inception to October 12, 2019, without language restriction, to find all potential publications. The following terms including “bivalirudin”, “extracorporeal membrane oxygenation”, “ECMO”, “extracorporeal life support”, “ECLS”, and “heparin induced thrombocytopenia” were searched both in Medical Subject Headings (MeSH) and free texts. The reference lists of retrieved articles to identify additional correlated articles were also reviewed. The websites (http://clinicaltrials.gov/) was also searched for other completed or ongoing trials.

### Selection of Studies

Two authors (HZ and ML-Z) independently reviewed the retrieved literatures to identify their eligibility. The studies that (1) reported patients presenting with HIT; (2) reported patients who underwent ECMO; and (3) reported the outcome of bivalirudin anticoagulation were considered to satisfy the inclusive criteria. In order to comprehensively collected relevant studies, although the study included patients without HIT besides patients with HIT, the study would still be considered for inclusion. Similarly, the other two inclusive criteria were also expanded. Additionally, we excluded studies if the patient (1) was not an adult; (2) did not receive bivalirudin after the HIT; or (3) did not undergo ECMO. Disagreements were resolved by discussions or consensus.

### Data Extraction

Two authors (HZ and ML-Z) extracted data independently. The following information were extracted from retrieved articles: (1) author, year of publication, and study location; (2) study type and sample size; (3) patient characteristics (including age, gender); (4) ECMO characteristics (including indication, mode, and duration); (5) diagnostic method of HIT; (6) treatment characteristics (including dosage of bivalirudin, anticoagulant therapy target and duration); and (7) major clinical outcomes.

### Assessment of Quality

The risk of bias of included randomised controlled trials or retrospective observational trials assessed by two authors (HZ and ML-Z) using the Cochrane Risk of Bias Tool (Higgins et al., 2011), New-castle Ottawa Scale (NOS) (Wells et al., 2013), Study Quality Assessment Tools, Tool for evaluating the methodological quality of case reports and case series (Murad et al., 2018), respectively.

### Statistical Analysis

For quantitative analysis, data were synthesized with Microsoft or Excel (2019). Continuous variables were presented as mean ± standard deviation (SD) for normal distributed variables or median (interquartile range) for skewed distributed variables. Categorical variables were presented as crude numbers or percentages. Linear regression was applied to assess the relationship between bivalirudin dosage and ACT in the case. A *p*<0.05 was considered statistical significance. Potential reporting biases were evaluated by funnel plots using STATA version 13.1 (StataCorp, College Station, TX, USA).

## Results

### Linear Therapeutic Response Between Bivalirudin and ACT During ECMO

In the present case, bivalirudin observed a linear therapeutic response. The formulations between the dosage of bivalirudin and ACT are as follows:

- The current patient underwent ECMO with CRRT (**Figure 1B**): y=392.36x+181.24; R^2^ = 0.236, *p*<0.05
- The current patient underwent ECMO without CRRT (**Figure 1C**): y=553.51x+186.22; R^2^ = 0.6408, *p*<0.05

y: ACT(s), x: dosage of bivalirudin (mg/kg/h), R^2^: % of variance explained by the model, *p*<0.05: statistically significant relationship between the independent variable and the dependent variable.

The equations represented that as the dosage of bivalirudin increased, the ACT value would ascend, vice versa, and there was a good linear therapeutic response between bivalirudin dosage and ACT. According to the constants of two equations, the present patient undergoing CRRT needed lower dosage of bivalirudin to achieve same level of ACT compared with her discontinuing CRRT.

### Description of the Systematic Review

**Figure 3:**
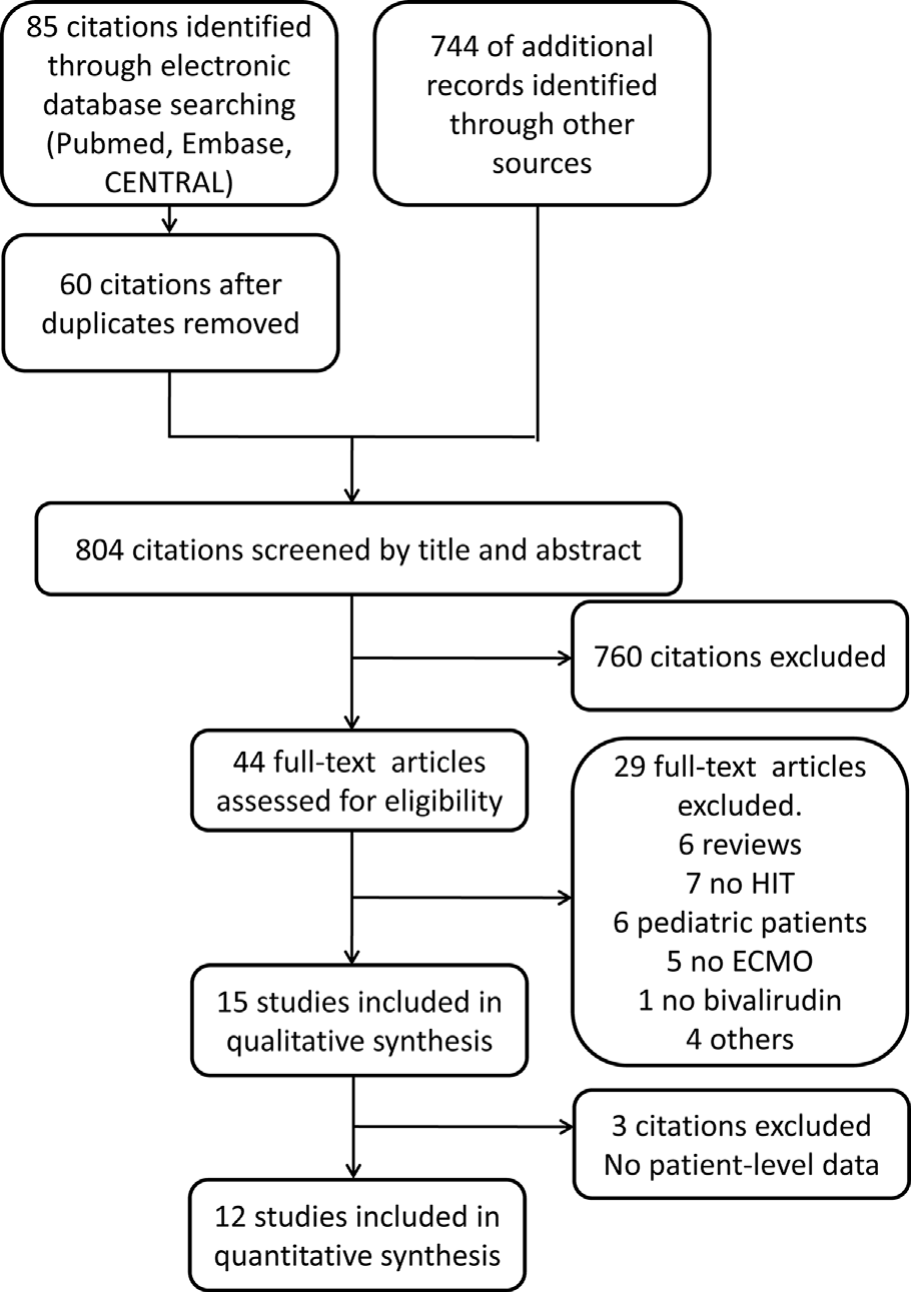
Flow diagram of studies those were assessed and included. CENTRAL: the Cochrane Central Register of Controlled Trials; HIT, heparin-induced thrombocytopenia; ECMO, extracorporeal membrane oxygenation.

Our search identified 829 articles including citations retrieved from Pubmed (28 citations), Embase (55 citations), Cochrane Library (2 citations), and 744 relevant references. After duplicates were removed, 804 articles were retrieved and screened by title and abstract. Afterwards, 44 citations were selected for full-text assessment. The excluded studies (29 citations) were reviews (6 studies), studies without HIT (7 studies), studies involving paediatric patients (6 studies), studies without ECMO (5 studies), studies without bivalirudin (1 study), and other exclusive criteria. Consequently, 15 articles were included for qualitative analysis, and 3 studies were excluded for quantitative analysis because the result of patient-level data could not be extracted from such studies. Finally, 12 studies were included for quantitative analysis (**Figure 3**).

### Methodological Quality of Included Studies

Most of the included studies were single arm retrospective observational studies or case reports. Therefore, risk of reporting biases could not be evaluated by funnel plots. According to the quality assessments, all of the studies observed good qualities (**Supplementary Tables 2**–**4**).

### Demographic Characteristics

The characteristics of studies reporting bivalirudin as an alternative anticoagulant for adult ECMO patients with HIT are summarised in **Table 1**. Seven of them were retrospective observational studies while eight were case reports. The inclusive studies for qualitative synthesis involved 123 patients, amongst whom 58 were confirmed or suspected with HIT, and 76 patients received bivalirudin as anticoagulant for ECMO. Most of the studies were conducted in USA (8 studies) (Pretzlaff et al., 2009; Atava et al., 2013; Bergh and Malhotra, 2013; Chen et al., 2017; Cremascoli et al., 2017; Natt et al., 2017; Klompas et al., 2019; Walker et al., 2019) and Europe [Germany 4 (Koster et al., 2007; Pappalardo et al., 2009; Koster et al., 2017; Ljajikj et al., 2017), Switzerland 1 (Pazhenkottil et al., 2016)], whereas one study was conducted in Chile (Van Sint Jan et al., 2017) and Egypt (Abdelbary et al., 2016) each.

**Table 1:** Characteristics of studies that reported bivalirudin as alternative anticoagulant for adult ECMO patients with HIT.

Reference	Location	Sample size	No. of patients with HIT	No. of patients receiving bivalirudin	ECMO indication	ECMO mode	ECMO duration (days)	Diagnosis of HIT	Loading bolus of bivalirudin [mg/kg]	Infusion of bivalirudin [mg/kg/h]	Target ACT(s)	Target aPTT (s)		Bivalirudin duration(days)	Main outcomes
**Retrospective observational study**															
Walker EA [2019] (Walker et al., 2019)	USA	14	11	14	ARDS: 12 CS: 2	VV	8 (range 1.5–28)	ELISA	0.2 mg/kg (not routinely administered)	0.15 (0.04 - 0.26); CRRT: 0.21 (0.09–0.36)	NA		1.5–2.5 x baseline	5.2 (0.9–28)	36% patients required a circuit change. 28.6% patients had bleeding. 64% patients survived to successful ECMO decannulation. 50% patients survived to hospital discharge.
VAn Sint Jan N [2017] (Van Sint Jan et al., 2017)	Chile	13	4	13	Cardiac and respiratory support	NA	31 ± 31	NA	None	0.08 ± 0.04	NA		NA	24 ± 33	Mortality: 43%. 2.7 ± 4 oxygenators were changed. No significant bleeding, thrombosis or circuit obstruction. Platelet count increased significantly.
NAtt B [2017] (Natt et al., 2017)	USA	5	5	4	RF, PAH	VV	9–50	PF4 AB, SRA	NA	NA	160 - 220		NA	2–47	Three survived and one death. Platelet counts recovered. No bleeding or thrombotic episodes.
Ljajikj E [2017] (Ljajikj et al., 2017)	Germany	57	21	21	LVAD implantation	VA	Intraoperate	ISCA	ACT < 160 s: 0.5; ACT > 160 s: 0.25	ACT< 160 s: 0.5; ACT> 160 s: 0.25	180–220		NA	Intraoperate	Low dose bivalirudin anticoagulation provides comparable results to UFH anticoagulation.
Abdelbary A [2016] (Abdelbary et al., 2016)	Egypt	12	2	2	RF, ECMO CPR	83% VV	12 (range 2–24)	NA	NA	0.1	NA		NA	NA	No bleeding or thrombotic complications related to bivalirudin. Platelet count recovered.
Atava A [2013] (Atava et al., 2013)	USA	7	7	7	LT	VV	NA	PF–4 AB, SRA	NA	NA	NA		NA	NA	Platelet count recovered.
Pretzlaff R [2009] (Pretzlaff et al., 2009)	USA	7 (5 adults)	NA	7	HF	NA	NA	NA	NA	Initial: 0.13 (0.05–0.22). The highest average dose: 0.47 (0.2–1.35).	NA		NA	204 h (range 49–670 h).	No patients died as a result of complications of their anticoagulation and no serious complications attributable to bivalirudin were recorded.
**Case report**															
Klompas A [2019] (Klompas et al., 2019)	USA	1	1	1	Aortic valve replacement	VA	17	PF4 AB	NA	NA	NA		80	15	Platelet counts improved, multiorgan failure worsened, and organ support was withdrawn.
Koster A [2017] (Koster et al., 2017)	Germany	1	1	1	COPD, LT	VV; VA	21	ISCA	None	Initial 0.2; Maintenance 0.1	167–203		NA	Intraoperate	Successful lung transplantation.
Cremascoli L [2017] (Cremascoli et al., 2017)	USA	1	1	1	CS	VA	13	PF4 AB	NA	NA	160 s		NA	8	Platelet count improved with no side effects.
Chen E [2017] (Chen et al., 2017)	USA	1	1	1	HF	VA	13	ELISA; SRA	0.75	1.75	2.5 x baseline		NA	≥ 13	Successful cardiac transplantation. Survived.
Pazhenkottil AP [2016] (Pazhenkottil et al., 2016)	Switzerland	1	1	1	HF	VA	NA	ELISA	NA	0.06	NA		1.5–2.5 x baseline	≥ 60	A large thrombus in the left main artery. Survived.
Bergh CC [2013] (Bergh and Malhotra, 2013)	USA	1	1	1	HF	VA	NA	PF4 AB	NA	NA	NA		NA	NA	Platelet count recovered above 50 x 109/L. Successfully removed from cardiovascular support.
Pappalardo F [2009] (Pappalardo et al., 2009 **)**	Germany	1	1	1	CS	VA	6	HIPA; ELISA; F1, 2; TAT; PGI	0.5	Initial 0.5; Maintenance 0.05 to 0.15	180–220		NA	8	Platelet counts increased. Survived.
Koster A [2007] (Koster et al., 2007)	Germany	1	1	1	HF, RVAD implantation	NA	7	PGI; HIPA	0.5 RVAD implantation:0.25	0.5 RVAD implantation: 1	200 to 220 RVAD implantation: 300–350		NA	38 h	Platelet counts increased to 45,000/nL. Survived.

ACT, activated clotting times; aPTT, activated partial thromboplastin time; ARDS, acute respiratory distress syndrome; COPD, chronic obstructive pulmonary disease; CS, cardiogenic shock; ECMO, extracorporeal membranous oxygenation; ELISA, anti-PF4/heparin antibodies enzyme linked immunosorbent assay; F1, 2, prothrombin fragment 1,2; H/B, Heparin/Bivalirudin; HF, heart failure; HIPA, heparin-induced platelet aggregation assay; HIT, heparin-induced thrombocytopenia; ISCA, IgG-specific chemiluminescent assay; LT, lung transplantation; LVAD, left ventricular assist device; NA, not available; PAH, pulmonary arterial hypertension; PF–4, platelet factor 4; PF4 AB, anti-PF4/heparin antibodies; PGI, Particle GEL immune assay; RF, respiratory failure; RVAD, right ventricular assist device; SRA, serotonin release assays; TAT, thrombin-antithrombin complex; VA, venoarterial; VV, venovenous.

Twelve studies were included for quantitative synthesis which consisted of 4 retrospective studies and 8 case reports. The patient-level data were extracted for analysis (**Supplementary Table 1**). As summarised in **Table 2**, 46 patients were retrieved. The mean age of patients with HIT receiving bivalirudin for ECMO anticoagulation was 46 years. Amongst them, 30 patients were males, and the gender of 2 patients was not shown.

**Table 2:** Summary of descriptive statistics of the patient-level data.

Variable	Patient-level data(n=46)
Age, average years	46.0
Male, n	30
Maintenance rate of bivalirudin, mean ± standard deviation (SD) (mg/kg/h)	0.27 ± 0.37
CRRT	0.15 ± 0.06
Without CRRT	0.28 ± 0.36
Duration of Bivalirudin (days)	8.58 ± 10.8
Platelet count on admission (/μl)	118900 ± 54500
Nadir of platelet count (/μl)	36700 ± 28552
Receiving platelet (units)	2.6 ± 1.5
Survived	29
Major bleeding	1
Thrombosis	4
Time of Platelet recovering (days)	3.3 ± 2.8

CRRT, continuous renal replacement therapies.

### ECMO Profiles

The indications of ECMO included heart failure (5 studies) (Koster et al., 2007; Pretzlaff et al., 2009; Bergh and Malhotra, 2013; Pazhenkottil et al., 2016; Chen et al., 2017), cardiac shock (3 studies) (Pappalardo et al., 2009; Cremascoli et al., 2017; Walker et al., 2019), respiratory failure (3 studies) (Abdelbary et al., 2016; Natt et al., 2017; Walker et al., 2019), lung transplantation (2 studies) (Atava et al., 2013; Koster et al., 2017), and others. The types of ECMO consisted of VV ECMO (5 studies) (Atava et al., 2013; Abdelbary et al., 2016; Koster et al., 2017; Natt et al., 2017; Walker et al., 2019) and VA ECMO (8 studies) (Pappalardo et al., 2009; Bergh and Malhotra, 2013; Pazhenkottil et al., 2016; Chen et al., 2017; Cremascoli et al., 2017; Koster et al., 2017; Ljajikj et al., 2017; Klompas et al., 2019). Overall, the duration of ECMO was ranged from 2 to 50 days (**Table 1**).

### Diagnosis of HIT

Platelet counts on admission were 118,900 ± 54,500/μl (mean ± SD). Nadir of platelet count was 36,700 ± 28,552/μl (mean ± SD) (**Table 2**). The HIT were confirmed by anti-PF4/heparin antibody analysis including enzyme linked immunosorbent assay (ELISA) and IgG-specific chemiluminescent assay (ISCA) (11 studies) (Pappalardo et al., 2009; Atava et al., 2013; Bergh and Malhotra, 2013; Chen et al., 2017; Cremascoli et al., 2017; Koster et al., 2017; Ljajikj et al., 2017; Natt et al., 2017; Klompas et al., 2019; Walker et al., 2019) and serotonin release assays (SRA) (3 studies) (Atava et al., 2013; Chen et al., 2017; Natt et al., 2017), and other platelet function tests (2 studies) (Koster et al., 2007; Pappalardo et al., 2009) (**Table 1**).

### Bivalirudin Regimens

The loading dose of bivalirudin was not routinely administered, and only 5 studies reported a loading dosage between 0.2 mg/kg to 0.75 mg/kg. The maintenance infusion dosages of bivalirudin were variable, from 0.05 mg/kg/h to 1.75 mg/kg/h. The average maintenance rate of bivalirudin was 0.27 ± 0.37 mg/kg/h. Additionally, the bivalirudin doses in patients with CRRT and patients without CRRT were 0.15 ± 0.06 mg/kg/h vs 0.28 ± 0.36 mg/kg/h, respectively (*p*=0.15) (**Table 2**). ACT and APTT were monitored in 7 and 3 studies respectively. The target of ACT ranged from 160 s to 220 s, while the target of APTT ranged from 45 s to 80 s. The longest duration of bivalirudin usage was more than 60 days (**Table 1**).

### Clinical Outcomes

The outcomes were platelet count recovery in 9 studies (Koster et al., 2007; Pappalardo et al., 2009; Atava et al., 2013; Bergh and Malhotra, 2013; Abdelbary et al., 2016; Cremascoli et al., 2017; Natt et al., 2017; Van Sint Jan et al., 2017; Klompas et al., 2019), bleeding or thrombosis in 5 studies (Abdelbary et al., 2016; Pazhenkottil et al., 2016; Natt et al., 2017; Van Sint Jan et al., 2017; Walker et al., 2019), mortality in 10 studies (Koster et al., 2007; Pappalardo et al., 2009; Pretzlaff et al., 2009; Pazhenkottil et al., 2016; Chen et al., 2017; Natt et al., 2017; Van Sint Jan et al., 2017; Klompas et al., 2019; Walker et al., 2019), and need to change oxygenator or circuit in 2 studies (Van Sint Jan et al., 2017; Walker et al., 2019). Most of the patients with confirmed HIT can improve platelet counts in 3.3 ± 2.8 days after switching to bivalirudin anticoagulation. A majority of cases (29 cases) survived, with 1 case reporting major bleeding and 4 cases reporting thrombotic events (**Table 2**).

## Discussion

In the current case, we reported a female who underwent ECMO with HIT complication and received bivalirudin as alternative anticoagulant. The platelet count recovered gradually. Meanwhile, the ACT maintained between 200 s to 250 s during the infusion of bivalirudin at an extremely low infusion rate of 0.005–0.15 mg/kg/h. Then, the ECMO was successfully weaned off without bleeding and thrombotic events.

### The Efficacy of Bivalirudin as ECMO Anticoagulant

The efficacy of bivalirudin has been extensively confirmed. The MATRIX trial randomized 7,213 acute coronary syndrome patients to bivalirudin or UFH treatments, and absolute benefits with bivalirudin were greater than UFH in patients who are vulnerable to hemodynamic or electrical disorders (Gargiulo et al., 2020). In the present case, bivalirudin successfully prevented thrombotic events during the entire course of ECMO run. ACT and APTT were kept within therapeutic ranges for most of the time. Meanwhile, the integrated review implies that the frequencies of clotting in patients treated with bivalirudin have been similar to those with UFH (Ranucci et al., 2011; Pieri et al., 2013; Berei et al., 2018). In addition, Rivosecchi RM, et al. has reported that bivalirudin reached therapeutic levels faster than UFH (30 vs. 48 h, *p*= 0.03), and it maintained levels in the therapeutic range more frequently than UFH (Rivosecchi et al., 2018). Ranucci M, et al. has shown that patients with bivalirudin treatment demonstrated significantly longer ACT, APTT, and reaction times at thromboelastography when compared to the UFH treatment (Ranucci et al., 2011).

### The Safety of Bivalirudin for Patients on ECMO With HIT

The safety of bivalirudin has been well elucidated. It has been reported that there is no risk of HIT from bivalirudin and other direct thrombin inhibitors (DTIs) (Di Nisio et al., 2005). The present case also reports that the platelet count increased to above 50x10^9^/L two days after initiation of bivalirudin therapy. The systematic review shows result similar to previous studies that the patients undergoing ECMO with HIT can improve platelet counts rapidly when switching to bivalirudin anticoagulation. Taking haemorrhage into consideration, the present patient and almost all the included case reports indicate no significant bleeding events induced by bivalirudin. Meanwhile, the pooled data showed that the incidences of bleeding events in patients with bivalirudin anticoagulation are comparable to those of UFH (Ranucci et al., 2011; Pieri et al., 2013; Berei et al., 2018). Likewise, the mortality by bivalirudin for ECMO patients with HIT is similar to the mortality of UFH in ECMO patients without HIT (Ranucci et al., 2011; Pieri et al., 2013; Berei et al., 2018). Overall, bivalirudin is a promising alternative anticoagulant agent to mitigate the pitfalls of UFH.

### The Titration of Bivalirudin During ECMO

Bivalirudin directly blocks thrombin without the cofactors. Approximately 80% of the drug is metabolised by proteolysis, while the remaining active drug is eliminated by the kidney unmetabolised (Bates and Weitz, 2000). These reasons suggest predictable correlation between bivalirudin dosage and anticoagulant efficacy (Netley et al., 2018). In the present case, the linear regression assessment showed a good linear therapeutic response between bivalirudin and ACT. Therefore, the titration of bivalirudin is feasible resulting in easy control of the target ACT or APTT in the present case.

As the renal function can affect the elimination of bivalirudin, it plays an important role in the bivalirudin dosing (Bates and Weitz, 2000). Tsu LV Walker and colleagues have demonstrated that patients with kidney dysfunction needed lower bivalirudin doses to achieve anticoagulant target. However, bivalirudin can be cleared by haemofiltration. It was documented bivalirudin doses were lower in patients with CRRT than those with normal nephritic function (creatinine clearance [CrCl] >60 ml/min), but higher than patients with renal impairment (CrCl <30 ml/min) and those not receiving CRRT (0.07 vs 0.13 vs 0.05 mg/kg/h, respectively; *p*<0.001) (Tsu and Dager, 2011). Furthermore, Pieri M and colleagues have displayed that adult ECMO patients undergoing CRRT required greater doses than those not on dialysis [0.041 (0.028–0.05) vs 0.028 (0–0.041) mg/kg/h, respectively (*p*=0.2) (Pieri et al., 2013). Similarly, Walker EA and colleague have reported that patients with CRRT required higher maintenance dose of bivalirudin than those without CRRT (0.21–0.36 vs 0.04–0.26 mg/kg/h, respectively) (Walker et al., 2019). However, the pooled data of present systematic review have shown non-significant difference of bivalirudin dosage between patients with CRRT or without CRRT. On the contrary, in the present patient, extremely low dose of bivalirudin has been used during CRRT because of previous minor bleeding in airways. The ACT has maintained between 160 s and 220 s. A major issue of DTIs was “APTT-confounding”. It is documented that patients with complications influencing prothrombin (e.g., coagulopathy secondary to disseminated intravascular coagulation, liver dysfunction, haemodilution, consumption of coagulation factors XI and XII on extracorporeal circuits) might show a false high APTT, which may lead to drug underdosing (Selleng and Selleng, 2016). We suspected that when ECMO was started on the present patient, the patient was acutely ill, and coagulopathic, thus the APTT and ACT increase was not only as a result of the bivalirudin dosing, but also because of associated critical illness coagulopathy.

After the withdrawal of CRRT, the bivalirudin dose has been titrated from 0.03 mg/kg/h to 0.15 mg/kg/h to maintain the target ACT. In addition, the systematic study has displayed a large variety of bivalirudin dose from 0.05 mg/kg/h to 1.75 mg/kg/h with or without loading dose. A lot of variables such as anticoagulation target, indication of ECMO, institution guideline, renal function, and so on may contribute to the heterogeneity (Sanfilippo et al., 2017). The optimal dosage of bivalirudin in adult patients under ECMO with HIT is not unified yet, suggesting careful dosing and monitoring of this unique population.

### The Management Recommendation of Bivalirudin for Patients With HIT During ECMO

**Figure 4:**
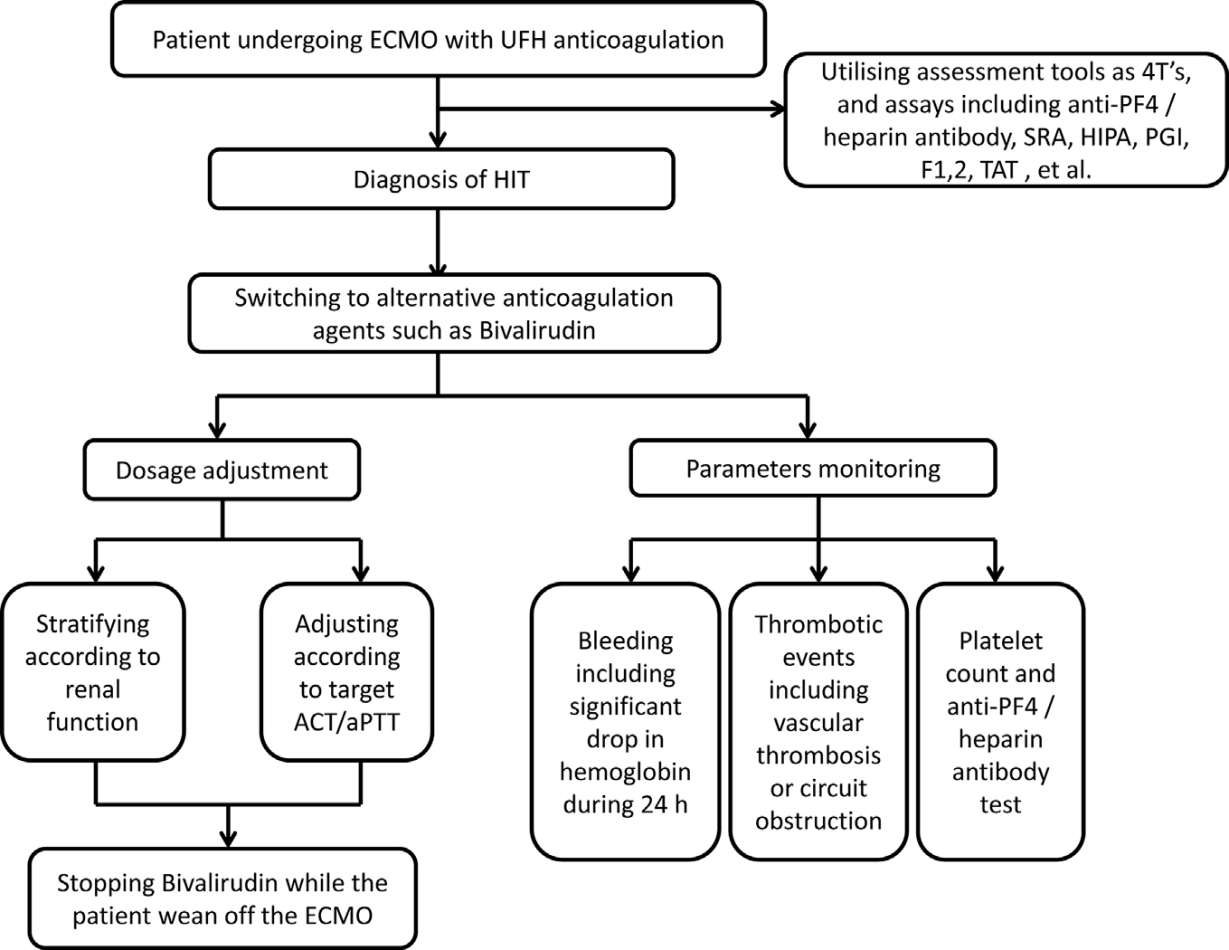
Management strategy for bivalirudin in adult ECMO patients with HIT. ECMO, extracorporeal membrane oxygenation; HIT, heparin-induced thrombocytopenia; UFH, unfractionated heparin; PF4, platelet factor 4; SRA, serotonin release assays; HIPA, heparin-induced platelet aggregation assay; PGI, Particle GEL immune assay; F1, 2, prothrombin fragment 1,2; TAT, thrombin-antithrombin complex; ACT, activated clotting times; aPTT, activated partial thromboplastin time.

Considering the huge variability in bivalirudin treatment, it is necessary to standardise the management of bivalirudin anticoagulation during ECMO support. According to the documented literature and the current patient’s experience, we deliver a brief flowgram shown in **Figure 4**. Foremost, the early identification of HIT was suggested through assessment tools such as 4T’s score, anti-PF4/heparin antibody assay, SRA, and other platelet function assays. Thereafter, the UFH should be switched to alternative anticoagulant. Bivalirudin was a good salvage option. On the one hand, the renal function of patient should be accessed to confirm the initial dosage of bivalirudin with or without loading bonus. Subsequently, the dose of bivalirudin should be adjusted according to the target ACT or APTT. On the other hand, the efficacy and safety of the bivalirudin could be monitored including the bleeding and thrombotic events. Meanwhile, tests of platelet count and anti-PF4 antibody could be repeated at the follow-up. Finally, the bivalirudin could be discontinued while the ECMO was weaned off. The ACT would normalise rapidly without reversal agent.

Currently, controlled trials comparing bivalirudin with other anticoagulants during ECMO are limited. Fortunately, two ongoing studies (NCT03707418 and NCT03965208) which investigated the efficacy and safety of bivalirudin compared to UFH in adult patients undergoing ECMO were ongoing to fulfil this gap. However, results have not been available yet. The upcoming results of these two randomised controlled studies may contribute to the precise use of bivalirudin.

### Limitations

Some limitations of the present study should be addressed. Firstly, most of the retrieved articles were observational studies without control group or case reports. Therefore, the meta-analysis of the pooled data was impossible. Secondly, the potential for publication bias should not be neglected as the negative results were harder for publication. Thirdly, we were not able to analyse the relationship between the demographic characteristics and dosage as a result of no comparator in almost all included studies. Fourthly, the dosages of bivalirudin in different studies were variable. It was tough to deliver a standard recommendation for ECMO adult populations. Finally, we suspected a selection bias because of the properties of the inclusive studies and small sample size.

## Conclusions

We report the first Chinese patient with HIT who used bivalirudin as anticoagulant during ECMO and successfully weaned from ECMO. We also firstly specify management path of ECMO patients’ anticoagulation with devastating complication. The systematic review showed that bivalirudin was a promising optimal choice for ECMO anticoagulation after HIT with good efficacy and safety. However, the large variability of the optimal dosage of bivalirudin was noteworthy in adult patients with ECMO. In addition, the lack of large sample study on this population with HIT during ECMO was obvious. In the future, prospective larger studies would be necessary to support the standard therapy of bivalirudin.

## Data Availability Statement

The datasets presented in this article are not readily available because this is a case report. Requests to access the datasets should be directed to guzhichun213@163.com.

## Ethics Statement

Ethical review and approval was not required for the study on human participants in accordance with the local legislation and institutional requirements. The patients/participants provided their written informed consent to participate in this study. Written informed consent was obtained from the individual(s) for the publication of any potentially identifiable images or data included in this article.

## Author Contributions

Z-CG and YG are the guarantors of the entire manuscript. HZ, M-LZ and Y-TY contributed to the study conception and design, critical revision of the manuscript for important intellectual content, and final approval of the version to be published. WL, S-PX, X-YZ, and W-JW contributed to the data acquisition, analysis, and interpretation.

## Funding

This study was funded by WU JIEPING medical foundation (320.6750.2020-04-31), Research Funds of Shanghai Health and Family Planning commission (20184Y0022, 20194Y0007), Cultivation fund of clinical research of Renji hospital (PY2018-III-06), Clinical Pharmacy Innovation Research Institute of Shanghai Jiao Tong University School of Medicine (CXYJY2019ZD001, CXYJY2019QN004), and Program for Key but Weak Disciplines of Shanghai Municipal Commission of Health and Family Planning (2016ZB0304).

## Conflict of Interest

The authors declare that the research was conducted in the absence of any commercial or financial relationships that could be construed as a potential conflict of interest.
