# Supplementary material for: Management of Bivalirudin Anticoagulation Therapy for Extracorporeal Membrane Oxygenation in Heparin-Induced Thrombocytopenia: A Case Report and a Systematic Review
Source: Front Pharmacol. 2020 Sep 11;11:565013. doi: 10.3389/fphar.2020.565013 (PMC7516194; doi:10.3389/fphar.2020.565013)
Supplement: Supplementary file 1 [file DataSheet_1.pdf]

Supplementary Table 1. The patient-level data including characteristics and outcomes of included studies.

Reference	No. of patients with HIT	Sequence of HIT patients	Age	Gender	Indication for ECLS	Mode of ECLS	Duration of ECLS (days)	Renal function	Maintenance rate of bivalirudin (mg/kg/h)	Duration of bivalirudin (days)	Platelet count on admission (μL)	Nadir of platelet count (μL)	Receiving platelet (units)	Survived	Major bleeding	Thrombosis	Time of platelet recovering (days)
**Retrospective observational study**																	
**Walker EA [2019] [12]**	11	1	36	F	ARDS	VV	28.125	77mL/min	0.13	28.4	NA	NA	NA	NA	NA	NA	NA
		2	21	M	ARDS	VV	3.666667	CRRT	0.2	2.6	NA	NA	NA	NA	NA	NA	NA
		3	20	F	ARDS	VV	5.416667	CRRT	0.22	3.4	NA	NA	NA	NA	NA	NA	NA
		4	27	M	ARDS	VV	1.75	60mL/min	0.13	1.6	NA	NA	NA	NA	NA	NA	NA
		5	47	M	ARDS	VV	10.75	CRRT	0.09	9.2	NA	NA	NA	NA	NA	NA	NA
		6	45	M	ARDS	VV	16.95833	iHD	0.21	7.7	NA	NA	NA	NA	NA	NA	NA
		7	51	F	ARDS	VV	5.25	57mL/min	0.1	5.2	NA	NA	NA	NA	NA	NA	NA
		8	36	M	ARDS	VV	19.54167	85mL/min	0.22	2	NA	NA	NA	NA	NA	NA	NA
		9	18	M	ARDS	VV	15.83333	210mL/min	0.26	0.9	NA	NA	NA	NA	NA	NA	NA
		10	46	M	Cardiac, post aortic root replacement surgery	VA	4.083333	CRRT	0.13	3.7	NA	NA	NA	NA	NA	NA	NA
		11	78	M	Cardiac, post-CABG	VA	6.625	12mL/min	0.04	2.9	NA	NA	NA	NA	NA	NA	NA

**Natt B [2017] [21]**	5 (1 argatroban, 4 bivalirudin)	1	41	M	hypoxemia	VV	30	NA	NA	16	129000	45000	3	Yes	No	No	NA
		2	26	F	pneumonia	NA	9	CRRT	NA	NA	NA	NA	1	Yes	No	No	NA
		3	41	M	ARDS	NA	13	NA	NA	2	144,000	77,000	NA	Yes	NA	NA	2
		4	32	F	H1N1 influenza	NA	50	NA	NA	47	128,000	98,000	NA	No	No	Extensive arterial and venous thrombosis	NA
**Ljajikj E [2017] [27]**	21		51.0 ± 12.7	16 (76.2%) M	LVAD implantation	VA	Intraoperate	Creatinine 1.68 ± 0.96 mg/dl	ACT < 160 s: 0.5; ACT > 160 s: 0.25	Intraoperate	NA	NA	2 (2–4)	19	1	NA	NA
**Abdelbary A [2016] [32]**	2		NA	NA	NA	VV	NA	NA	0.1	NA	NA	NA	NA	NA	NA	NA	2d
**Case report**																	
**Klompas A [2019] [20]**	1		66	F	Aortic valve replacement	VA	17	CRRT	NA	15	63000	21000	5	No	NA	NA	NA
**Koster A [2017] [28]**	1		58	M	COPD, LT	VV; VA	21	NA	Initial 0.2; Maintenance 0.1	Intraoperate	152000	47000	3	Yes	NA	NA	NA

**Cremascoli L [2017] [22]**	1		60	M	CS	VA	13	NA	NA	8	229000	16000	NA	Yes	NA	NA	8
**Chen E [2017] [23]**	1		55	M	HF	VA	13	NA	1.75	≥ 13	NA	NA	NA	Yes	NA	Femoral and the axillary artery access sites thrombus.	NA
**Pazhenkottil AP [2016] [30]**	1		62	M	HF	VA	NA	Renal insufficiency requiring dialysis	0.06	≥ 60	138000	24000	NA	Yes	NA	A large thrombus in the left main artery.	NA
**Bergh CC [2013] [24]**	1		69	F	HF	VA	NA	NA	NA	NA	16000	9000	NA	Yes	NA	NA	0.5
**Pappalardo F [2009] [29]**	1		71	F	CS	VA	6	Renal function recovered	Initial 0.5; Maintenance 0.05 to 0.15	8	110000	10000	NA	Yes	NA	Intracardiac thrombus formation	5
**Koster A [2007] [10]**	1		40	F	HF, RVAD implantation	NA	7	NA	0.5 RVAD implantation: 1	38 hours	80000	20000	1	Yes	NA	NA	1

ACT: activated clotting times; ARDS: acute respiratory distress syndrome; COPD: chronic obstructive pulmonary disease; CS: cardiogenic shock; ECMO:extracorporeal membranous oxygenation; ELISA: anti-PF4/heparin antibodies enzyme linked immunosorbent assay; F: female; HF: heart failure; HIT: heparin-induced thrombocytopenia; LT: lung transplantation; LVAD: left ventricular assist device; M: male; NA: not available; RVAD: right ventricular assist device; VA: venoarterial; VV: venovenous.

Supplementary Table 2. Methodological quality of case series: the review authors' judgments about each quality item for each included case series.

Criteria	Reference					
	Walker EA [2019]	Van Sint Jan N [2017]	Natt B [2017]	Abdelbary A [2016]	Atava A [2013]	Pretzlaff R [2009]
1. Was the study question or objective clearly stated?	Yes	Yes	Yes	Yes	Yes	Yes
2. Was the study population clearly and fully described, including a case definition?	Yes	Yes	Yes	Yes	Yes	Yes
3. Were the cases consecutive?	Yes	Yes	Yes	Yes	Yes	Yes
4. Were the subjects comparable?	Yes	Yes	Yes	Yes	Yes	Yes
5. Was the intervention clearly described?	Yes	Yes	Yes	Yes	Yes	Yes
6. Were the outcome measures clearly defined, valid, reliable, and implemented consistently across all study participants?	Yes	Yes	Yes	Yes	Yes	Yes
7. Was the length of follow-up adequate?	Yes	Yes	Yes	Yes	Yes	Yes
8. Were the statistical methods well-described?	No	Yes	No	No	No	No
9. Were the results well-described?	Yes	Yes	Yes	Yes	Yes	Yes
Quality Rating	Good	Good	Good	Good	Good	Good

Supplementary Table 3. Methodological quality of case-control studies: the review authors' judgments about each quality item for each included case-control study.

Criteria	reference

	Ljajikj E [2017]
1. Was the research question or objective in this paper clearly stated and appropriate?	Yes
2. Was the study population clearly specified and defined?	Yes
3. Did the authors include a sample size justification?	Yes
4. Were controls selected or recruited from the same or similar population that gave rise to the cases (including the same timeframe)?	Yes
5. Were the definitions, inclusion and exclusion criteria, algorithms or processes used to identify or select cases and controls valid, reliable, and implemented consistently across all study participants?	Yes
6. Were the cases clearly defined and differentiated from controls?	Yes
7. If less than 100 percent of eligible cases and/or controls were selected for the study, were the cases and/or controls randomly selected from those eligible?	NA
8. Was there use of concurrent controls?	Yes
9. Were the investigators able to confirm that the exposure/risk occurred prior to the development of the condition or event that defined a participant as a case?	Yes
10. Were the measures of exposure/risk clearly defined, valid, reliable, and implemented consistently (including the same time period) across all study participants?	Yes
11. Were the assessors of exposure/risk blinded to the case or control status of participants?	No
12. Were key potential confounding variables measured and adjusted statistically in the analyses? If matching was used, did the investigators account for matching during study analysis?	Yes
Quality Rating	Good

NA: Not applicable.

Supplementary Table 4. Methodological quality of case reports: the review authors' judgments about each quality item for each included case report.

Criteria	Reference

	Klompas A [2019]	Koster A [2017]	Cremascoli L [2017	Chen E [2017]	Pazhenkottil AP [2016]	Bergh CC [2013]	Pappalardo F [2009]	Koster A [2007]
1. Does the patient(s) represent(s) the whole experience of the investigator (centre)?	Yes	Yes	Yes	Yes	Yes	Yes	Yes	Yes
2. Was the exposure adequately ascertained?	Yes	Yes	Yes	Yes	Yes	Yes	Yes	Yes
3. Was the outcome adequately ascertained?	Yes	Yes	Yes	Yes	Yes	Yes	Yes	Yes
4. Were other alternative causes that may explain the observation ruled out?	Yes	Yes	Yes	Yes	Yes	Yes	Yes	Yes
5. Was there a challenge/rechallenge phenomenon?	No	No	No	No	No	No	No	No
6. Was there a dose–response effect?	No	No	No	No	No	No	No	No
7. Was follow-up long enough for outcomes to occur?	Yes	Yes	Yes	Yes	Yes	Yes	Yes	Yes
8. Is the case(s) described with sufficient details to allow other investigators to replicate the research or to allow practitioners make inferences related to their own practice?	Yes	Yes	Yes	Yes	Yes	Yes	Yes	Yes
Quality Rating	Good	Good	Good	Good	Good	Good	Good	Good
